# General Medicine and Therapeutics

**Published:** 1893-11

**Authors:** 


### GENERAL MEDICINE AND THERAPEUTICS.

EDITED BY DR. LUTHER B. GRANDY.

### GUAIACOL IN TYPHOID FEVER.

Having read in a recent copy of the New York *Herald* the experience of M. Holscher, of Mulheim, with this drug in treating sixty cases of typhoid fever, I thought perhaps some good might come from a brief account of my experience with this agent in treating a few cases within the last few months.

Some three months ago I had in charge a man thirty-five years of age, suffering from a mild attack of typhoid, who, after having had no abnormal temperature on the twenty-first and twenty-second days, suddenly had the temperature to go up to 100° F. at five P. M. on the twenty-third day. On questioning him, I found that ten years previous to that time he had suffered from what he termed bronchial hemorrhages, and although I found no signs of either recent or old chest trouble on physical examination, yet there was slight cough and scanty mucous expectoration, and I therefore thought it best to put him on guaiacol in five drop doses, three times a day. After forty-eight hours the temperature fell to normal, but the cough and expectoration continued. This rapid effect of the drug upon the temperature made me think at once of the possibility of its having the power of arresting the growth of the typhoid bacilli in the intestine.

Since then I have treated seven cases of typhoid with it, and my friend, Dr. C. F. Newbill, at my suggestion, has tested its efficacy in twelve other cases, making in all nineteen cases.

Most of the cases have been mild types of the disease, with few complications and no violent symptoms, and they have varied greatly in ages, from a baby of eighteen months to an old lady of sixty-five years.

As our German brother claims, we have found no need of antipyretic measures of any sort, and the temperature is kept constantly low, simply by the administration of this drug. With it the temperature falls, the pulse grows less rapid, the tympanites and diarrhea disappear, the tongue becomes moist and clean, the nervous symptoms rapidly subside, and the patient goes on to a complete recovery. This has been proven in each of the nineteen cases treated by us.

It seems to me that this is sufficient trial to justify further investigation of the power of the drug in controlling the fever in these cases, and I send you this mention of my experience, hoping that you will see fit to publish it, and that some of my *confrères* in the profession may adopt my suggestion, and after trying it, will write us the results of their experience with the drug in similar cases.—Dr. Baker in *N. Y. Medical Record.*

---

### SYPHILITIC SPINAL PARALYSIS.

Oppenheim (*Berl. klin. Woch.*, August 28, 1893) refers to the syphilitic spinal paralysis recently described by Erb. The gait is stiff-legged, although there is relatively little muscular rigidity, the tendon reflexes are increased, but the motor loss is not so great as the gait would lead one to expect. Unlike ordinary spastic paralysis, there is almost constant weakness of the bladder, diminished sexual power, and slightly marked sensation troubles. The condition develops in the course of months or years, or sometimes more rapidly. Sometimes there is great variation in the symptoms. Improvement may occur after inunction. The patients mostly do not become paraplegic, as in transverse myelitis, or if they do, the paralysis improves. Erb thinks it due to a partial horizontal lesion in the dorsal cord. The author says that myelitis plays an important part in the clinical history of spinal syphilis. It has a tendency to improve, to get well, or it may remain stationary. Recent researches in spinal syphilis have shown that the chief form consists in a meningo-myelitis, the lesion starting in the membranes. Erb thinks that the disease described by him has nothing to do with meningitis, but the author would look upon it as a relatively favorable form of this meningo-myelitis, more or less localized in the dorsal region. The meningeal affection, as well as the changes in the cord, so far as they are syphilitic, may clear up, and only the after results remain, namely the myelitis, and especially secondary degenerations. Pathological anatomy has demonstrated the lesion limited to the dorsal cord. Thus the author would look upon this syphilitic spinal paralysis as a stage or a special localization in this meningo-myelitis. Only a few cases correspond to Erb's description, for they mostly show more extensive symptoms. The author hardly regards the prognosis as very favorable. The symptoms are not absolutely characteristic, but the fluctuating course of the disease, the effect of treatment, and the existence of (1) present or past cerebral symptoms, (2) meningeal irritation symptoms, (3) manifestations pointing to several foci of disease, or (4) the undeveloped symptom complex of Brown-Sequard's paralysis, are more definite guides to the differential diagnosis.—*British Medical Journal.*

---

### COMPARATIVE INFLUNCE OF LARGE AND SMALL DOSES OF IRON.

The comparative effects of large and small does of iron and the indications for the one or the other are discussed in the *Therapeutic Gazette.* The experience of the writer in treating twelve cases of anemia which were as far as possible alike, confirmed his belief in the value of small doses. Six of these people received two or three grains of reduced iron three times a day, and the remaining six received one-third of a grain three times daily. The six that received the small doses had far less disorder of digestion than those who received the large doses. They recovered as promptly as those receiving the large doses, if not more so. It is true that in some conditions in which there is gastro-intestinal disorder associated with the formation of gas arising from fermentation or decomposition, and in which the anemia is largely due to destruction of the constituents of the blood by the absorption of poisonous materials from the intestine, large doses of iron are absolutely necessary, because in these instances only a small quantity of iron is absorbed, and the greater amount of it forms a sulphide of iron, or other compound, with the contents of the intestine. Where we have, therefore, a destruction of the iron in large amount it may be necessary to give it in full doses; but unless this is the case one-eighth of a grain of reduced iron will in most cases give better results than three grains. Under these circumstances constipation more rarely occurs, and we also avoid in this way the so-called iron headache.—*Boston Medical and Surgical Journal.*

---

### The Feeding of Infants.

Hauser (*Berl. klin. Woch.*, August 14, 1893) describes a new method. He first refers to the well-known objections to a wet nurse, and the difficulties in artificial feeding. The author has used, in Henoch's clinic and elsewhere, a preparation introduced by Rieth, in which, after the smaller quantities of fat and sugar, in cow's milk have been corrected by the addition of cream and milk sugar, egg albumen, heated above 130° C., is made to supply the deficiency in albumen. The preparation has the same composition as woman's milk, and is called albumen milk ("Eiweissmilch"), but would be more correctly named albumose milk. The difference between this and ordinary milk, when subjected to artificial digestion, is obvious. If feeding with cow's milk properly prepared and sterilized does not suit, the author uses this preparation. Medicinal agents are not employed, and washing out the stomach is rarely necessary. There are two classes of cases, (1) those in which cow's milk properly prepared seems to suit, and yet the infants do not thrive, and (2) those with dyspepsia, etc. Some sixty infants were treated with this preparation, and the author has now used it for one year and a half. The infants take it well, vomiting ceases even in those in whom other preparations have failed, and the weight increases. It is given in small quantities, and in cold in bad cases. The stools become healthy and regular, but they may be offensive owing to the sulphur in the albumoses. Failure is rare. This preparation is also useful in acute illness, in rickets, and some other diseases of children. Infants with whom mother's milk does not agree take is well. Older infants also thrive on it. Cow's milk may be added to it until pure milk feeding is arrived at.

---

### CHLORATE OF SODA IN CANCER OF THE STOMACH.

At a meeting of the French Association for the Advancement of Science in August last, Dr. Brissaud, of Paris, made a report upon the above subject. He said that it was an established fact that solutions of chlorate of potash exert a very favorable influence on epithelioma of the mouth and certain forms of cancroid of the face. The successful results obtained with this method induced him to try the effect of the corresponding salt of sodium in the treatment of carcinoma of the stomach. This salt was selected because it is much less toxic and more soluble than chlorate of potash, seeing that relatively large doses may be injected into animals without ill effect, and that it is soluble in three times its own weight of water, while the potassium salt requires twenty parts of water for complete solution. He had treated several undoubted cases of cancer of the stomach by the administration of chlorate of sodium in doses varying from eight to sixteen grammes (two to four drachms) in the twenty-four hours. The results were so satisfactory that if his observations were limited to one or two cases only he would hesitate to publish them, because he might possibly have mistaken cases of chronic gastritis for cancer, for the former, as is well known, sometimes present all the symptoms of malignant disease; but during the last four years he had tried this treatment with equal success in five successive cases of localized gastric cancer, and it is difficult to admit that an error of diagnosis was committed in each of these five cases. This is the more improbable, seeing that three of the patients presented a distinct epigastric tumor. In every case the treatment was followed by the most remarkable improvement, amounting practically to a cure. The drug was given in doses of twelve, fourteen, or even sixteen grammes in the twenty-four hours, and the patients may now be regarded as radically cured.

Under the influence of this treatment, the melena, hematemesis and cachexia disappeared; the appetite returned; and in the three cases in which a tumor could be detected in the epigastrium, it gradually subsided until no trace of it was left at the end of six weeks. On the other hand, the treatment failed in a number of cases, for while chlorate of sodium seems to exert a favorable influence on malignant tumors of the epithelial type, it produces no effect whatever on the intestinal sarcomatous type of neoplasms. Moreover, the failure in some of these cases is to be attributed either to the fact that cancer had already spread to other organs, or to the presense of complications which were not amenable to the treatment. For example, in one case the liver was already involved when the administration of chlorate of soda was commenced. In another patient, a young woman who had at first experienced some relief from the treatment, the disease suddenly extended to other parts of the body, where it developed with extreme rapidity. She was a patient of Dr. Nélaton; she received sixteen grammes of chlorate of soda a day, and in the course of a few weeks hematemesis ceased, appetite returned, and she put on sixteen pounds in weight. This improvement was so remarkable as to suggest an error of diagnosis. At the autopsy, however, cancerous nodules were found in various organs and tissues. In a third case death was due to phlebitis of the inferior vena cava. It cannot, therefore, be included in the statistics of the treatment of gastric cancer by chlorate of soda.—*Medical Record.*

---

### SUMMER DIARRHEA.

℞. Bismuth subnit., ʒ ss.

Tr. opii., gtt. xx.

Syr. ipecac,

Syr. rhei arom., āā ʒ iv.

Listerine, ℥ ss.

Mist. cretæ, ℥ j.

M. Sig.: Teaspoonful once in three or four hours for a child ten or twelve months old.—*Roberts in Med. Record.*

### NON-BITTER QUININE.

℞. Quinine sulph., gr. xv.

Acid. sulph. dil., M xv.

Spr. menth. pip., ʒ ijss.

Sol. saccharin. saturat., ʒ v.

Aquæ dest., ℥ vj.

—*Medical News.*

---

### FOR MYALGIA.

R. Liniment chloroformi, } āā fʒ iv.

Liniment aconiti, }

Tinct. opii., fʒ iij.

Liniment saponis, ad f℥ iv.—M.

Ft. liniment.

S.: To be well rubbed into the painful parts.

—*The Practitioner.*

---

For *pain in the ear* from inflammation, Dr. John Dunn (quoted in *La Semaine Médicai*) recommends the following:

℞. Menthol pulv.,
Camphor pulv., āā gr. xx.
Vaseline liquid, f℥ j.
M. Sig.: Instill a few drops into the ears several times a day.

---

For *urticaria of children* (*L'Union Méd.*):

℞. Chloral hydrat.,
Camphoræ pulv.,
Acaciæ pulv., āā ʒ j.
Triturate until liquified, and add
Cerat. simpl., ʒ j.
M. Sig.: Apply topically.

—*Canada Medical Record.*

For *irritable cough* a writer in the *Practitioner* suggests:

℞. Acidi hydrocyanici diluti, f℥ iss.

Morphinæ acetatis, gr. iss.

Mucilaginis acaciæ, ℥ j

Syrupi pruni virginianæ, f℥ iv.

Aquam, ad. ℥ vj.

Misce et fiat mistura.

A teaspoonful to be sipped every four or six hours.

---

For the *night-sweats of pulmonary tuberculosis*, Dr. Ewart (*La Semaine Méd.* in *Med. News*) suggests:

℞. Quiniæ sulphat.,
Zinci sulphat., āā gr. ij.
Ext. hyoscyami, gr. j.
Ext. nucis vomicæ, gr. $\frac{1}{3}$.

M.

Ft. pil. j.

Sig.: Take at bedtime.

---

For an *emulsion of cod liver oil* (*The Practitioner*):

℞. Ol. morrhuæ, m xxx.
Glycerini, m x.
Liquor. calcis, vel
Mucilag. acaciæ, f℥ j.

M.

---

For the *insomnia of children*, Simon (*L'Union Méd.*, in *Therap. Gazette*) employs the following injection:

℞. Chloral, gr. ij.
Tinct. moschi, gtt. xx.
Tinct. valerian, gtt. xx.
Aquæ destillat., f℥ j.

M.

Inject the entire quantity into the rectum, and, if necessity requires it, the dose may be repeated if sleep does not come on in the course of two or three hours.

—*Canada Medical Record.*

